# Treatment of *Mycobacterium tuberculosis*-Infected Macrophages with Poly(Lactic-Co-Glycolic Acid) Microparticles Drives NFκB and Autophagy Dependent Bacillary Killing

**DOI:** 10.1371/journal.pone.0149167

**Published:** 2016-02-19

**Authors:** Ciaran Lawlor, Gemma O’Connor, Seonadh O’Leary, Paul J. Gallagher, Sally-Ann Cryan, Joseph Keane, Mary P. O’Sullivan

**Affiliations:** 1 School of Pharmacy, Royal College of Surgeons in Ireland, Dublin 2, Ireland; 2 Trinity Centre for Bioengineering, Trinity College Dublin, Dublin 2, Ireland; 3 Department of Clinical Medicine, Institute of Molecular Medicine, Trinity College Dublin, and St. James’ Hospital, Dublin, Ireland; The Catholic University of the Sacred Heart, Rome, ITALY

## Abstract

The emergence of multiple-drug-resistant tuberculosis (MDR-TB) has pushed our available repertoire of anti-TB therapies to the limit of effectiveness. This has increased the urgency to develop novel treatment modalities, and inhalable microparticle (MP) formulations are a promising option to target the site of infection. We have engineered poly(lactic-co-glycolic acid) (PLGA) MPs which can carry a payload of anti-TB agents, and are successfully taken up by human alveolar macrophages. Even without a drug cargo, MPs can be potent immunogens; yet little is known about how they influence macrophage function in the setting of *Mycobacterium tuberculosis* (Mtb) infection. To address this issue we infected THP-1 macrophages with Mtb H37Ra or H37Rv and treated with MPs. In controlled experiments we saw a reproducible reduction in bacillary viability when THP-1 macrophages were treated with drug-free MPs. NFκB activity was increased in MP-treated macrophages, although cytokine secretion was unaltered. Confocal microscopy of immortalized murine bone marrow-derived macrophages expressing GFP-tagged LC3 demonstrated induction of autophagy. Inhibition of caspases did not influence the MP-induced restriction of bacillary growth, however, blockade of NFκB or autophagy with pharmacological inhibitors reversed this MP effect on macrophage function. These data support harnessing inhaled PLGA MP-drug delivery systems as an immunotherapeutic in addition to serving as a vehicle for targeted drug delivery. Such “added value” could be exploited in the generation of inhaled vaccines as well as inhaled MDR-TB therapeutics when used as an adjunct to existing treatments.

## Introduction

Nearly 1.5 million people die of tuberculosis (TB) annually because current vaccines and therapeutic regimens are inadequate. The emergence of multiple-drug-resistant TB has stretched our existing therapeutics beyond their ability to deal with the epidemic. There is a pressing requirement to develop new drugs and modalities that may include patient-directed therapies, including immunotherapeutics. One promising option is to repurpose existing anti-TB agents as cargo in advanced drug delivery systems that could also facilitate pharmaceutical development of emerging therapeutics. One such method harnesses therapeutic aerosol bioengineering [1] to prepare inhalable microparticles (MPs). MPs manufactured, using well-established materials, such as the US Food and Drug Administration–approved material poly(lactic-co-glycolic acid) (PLGA), can deliver a drug cargo to the intracellular environment of phagocytes [2]. PLGA polymers are biodegradable and approved for use in a range of commercially marketed therapeutic products. While PLGA has been widely investigated for the preparation of drug-loaded particles for inhalation these have yet to make it to market. Much of the delay in translation of these very promising technologies lies in the lack of knowledge about the toxicological and immunogenic effects of PLGA particles once deposited in the lungs after inhalation.

The use of inhalable delivery systems to treat TB represents an appealing alternative to traditional oral/parenteral formulations. By localising anti-tubercular therapy to the lungs, systemic toxicity may be greatly decreased and with increasing local concentrations of anti-tubercular therapy the duration of treatment may be reduced also [3, 4]. These factors combined may help to improve patient compliance rates, thereby reducing the emergence of resistant strains. Inhalable anti-tubercular delivery systems based on polymeric MPs have shown great potential both *in vitro* and *in vivo* [5]. PLGA MPs are efficiently phagocytosed by human alveolar macrophages [6, 7] and we have seen potent microbicidal effects when they carry a payload of anti-TB drugs [8]. Although the macrophage is the effector cell in the host response to *Mycobacterium tuberculosis* (Mtb), it is also the niche cell for the bacillus; where Mtb can replicate before causing cell death and moving on to infect other cells [9]. Phagocytosis of MPs by this cell therefore provides an opportunity for targeted killing of intracellular bacilli. Such an inhaled approach might improve the effectiveness of anti-TB therapies by reducing the frequency of dosing and maximising local deposition of the anti- tubercular agent.

A key challenge in the development of inhaled MP therapies will be defining their effects, if any, on innate and adaptive immune responses in the lung. Although a number of studies have shown that PLGA MPs are inert [10–12] making them suitable candidates for inhalable therapy, others have demonstrated that PLGA based MPs can activate endogenous macrophage responses [13–18]. The immunogenicity of synthetic MPs is dependent on physical properties of the particle such as size, shape, composition, surface chemistry and electrical charge, as well as the ability of host cells to recognise them via the expression of appropriate cell surface receptors. In the presence of pathogen-associated molecular patterns (PAMPs), such as LPS, polymeric MPs can drive important phagocyte functions, such as IL-1β [19] and TNFα production [18] and antigen presentation [20]. Thus far some authors have suggested that they may also support macrophage responses to Mtb infection by stimulating cytokine production and modulation of cell death pathways [16, 17, 21].

In the present study we sought to characterise the immune phenotype of macrophages following treatment with drug-free MPs by evaluating established pathways for endogenous macrophage control of Mtb infection. To this end, we infected THP-1 macrophages with Mtb and monitored bacillary replication in the setting of drug-free MP treatment. We found that PLGA MPs, without a drug payload, can limit intracellular Mtb replication without altering the cytokine profile produced by macrophages infected with Mtb. Treatment of uninfected cells with MPs did not induce the secretion of pro-inflammatory cytokines or significantly alter the viability of the cells. However, MPs stimulated the activation of the NFκB pathway and autophagy in uninfected macrophages. Inhibition of NF- κB or autophagy with pharmacological inhibitors reversed the ability of MP-treated macrophages to limit Mtb growth.

## Materials and Methods

### Polylactide-co-glycolide (PLGA) Microparticle Manufacture and Characterisation

Polylactide-co-glycolide (PLGA) 503H (Boehringer Ingelheim, Germany) MPs were prepared using a double emulsion, solvent evaporation method [22]. Briefly, 50mg PLGA was dissolved in dichloromethane (2.5% w/v) and the solution was remotely probe sonicated. A primary emulsion (w_1_/o) was created by the addition of 2.5% w/v poly(vinyl) alcohol (PVA) followed by probe sonication for 16 seconds. A final secondary emulsion (w_1_/o/w_2_) was formed by transferring the primary emulsion into a continuous phase consisting of 20ml 1% PVA. Following homogenisation, the emulsion was mechanically stirred in the fume hood over-night to allow the solvent to evaporate and allow microparticle formation. Microparticles were then centrifuged at 7,000g for 7 minutes and washed in distilled water three times to remove residual PVA, prior to lyophilisation. Where required, the hydrophilic fluorescent dye tetramethylrhodamine isothiocyanate (TRITC) was added to the first aqueous phase of the emulsion above (10% w/w).

Microparticles were characterised for size by laser diffraction using a Mastersizer 2000 (Malvern) for surface charge by zeta-potential (Malvern Zetasizer) and for morphology by scanning electron microscopy (SEM) using a Tescan MIRA XMU, secondary electron detector at an electron voltage of 5.0Kv [7].

### Mycobacteria

*M*. *tuberculosis* strains H37Rv and H37Ra were obtained from the American Type Tissue Culture (Manassas, VA). Stocks were propagated in Middlebrook 7H9 medium (Difco/Becton Dickinson, Sparks, MD) made up in low-endotoxin water (Sigma, St. Louis, MO) supplemented with albumin-dextrose-catalase supplement (Becton Dickinson) and 0.05% Tween 80 (Difco). Aliquots were stored at –80°C, thawed and propagated in Middlebrook 7H9 medium to log phase before use.

### Cell culture and infection

Immortalised murine bone marrow derived macrophages (BMDM) which had been stably transfected with GFP-LC3 were a kind gift from Dr Hardy Kornfeld and Dr Michelle Hartman, University of Massachusetts, USA [23, 24] and were maintained in DMEM containing 5μg/ml puromycin and 10% fetal calf serum. The day before infection the cells were trypsinized and seeded into culture dishes and 2 well Labtek chamber slides (Nunc). During and after infection these cells were maintained in DMEM/10% fetal calf serum without puromycin.

THP-1 cells from the ATCC (TIB-202) were maintained in RPMI-1640 (GIBCO), supplemented with 10% foetal bovine serum (GIBCO). Prior to infection, the cells were seeded into culture dishes and 2-well Labteks at a density of 1x10^5^ cells /ml and differentiated using 100nM PMA for 72 hours.

On the day of infection, log-phase bacilli were centrifuged at 2,600 x *g* for 10 minutes and the bacilli were re-suspended in RPMI-1640 containing 10% foetal bovine serum. Clumps were dispersed by passing the bacilli through a 25-gauge syringe six to eight times and the sample was centrifuged at 100 x *g* for 3 minutes to sediment any remaining clumps of mycobacteria. To determine the amount of dispersed mycobacteria required to achieve the appropriate multiplicity of infection (MOI), macrophages in Labteks were treated with different amounts of mycobacteria and incubated for 3 hours at 37°C. Extracellular bacteria where then washed off using phosphate buffered saline and the macrophages were fixed in 2% paraformaldehyde for 5 minutes and stained for acid fast bacilli as previously described [25]. Briefly, the cells were stained with auramine-M (Becton Dickinson), washed in distilled water and treated with the TB-Declorizer ™. Hoescht 33342 (10μg/ml) was used to counter-stain cell nuclei and anti-fade medium (DAKO, Glostrup, Denmark) was added before imaging. The number of bacilli per cell was determined using an Olympus IX51 inverted fluorescent microscope (Olympus IX51, Olympus Europa GmbH, Germany). Based on this result macrophages in culture dishes were then infected to reach a MOI of approximately 1–5 phagocytosed bacilli per cell for 3 hours, and then washed to remove extracellular mycobacteria. The cells were then treated for 1 hour with 2.2μm PLGA MPs or 1.8 μm polystyrene (PS) MPs (Sigma) at a concentration of 200μg/ml (unless otherwise stated), washed and incubated at 37°C and 5% CO_2_ for a total of 72 hours. Infected cells were lysed and harvested for colony forming unit (CFU) enumeration on Middlebrook agar plates. Agar plates were incubated at 37°C and plates with a statistically relevant number of colonies were counted between 14 and 21 days and used to calculate the number of CFU per ml.

### Confocal laser scanning microscopy (CLSM)

Immortalised BMDMs expressing GFP-LC3 were seeded in 8-well coverslip Labtek chamber slides for live cell imaging on a confocal laser scanning microscope. The cells were treated with 2.2μm PLGA microparticles labelled with tetramethylrhodamine isothiocyanate (TRITC) and the induction of autophagy was monitored. In some experiments the cells were incubated with DQ-BSA for 2h to label lysosomes and then treated with blank (unstained) MP. For immunostaining, cells were fixed for 10 minutes with 2% paraformaldehyde (Sigma, St Louis, Mo), permeabilized for 10 minutes and incubated with antibodies to LAMP-1 (rat and anti-mouse, clone CD107a, eBioscience) and Polyclonal Anti-Mycobacterium tuberculosis Whole Cell Lysate minus LAM: NR13820 (obtained from BEI Resources NIAID, NIH) followed by goat-anti Rat-Alexa-594 and goat anti-rabbit-Alexa-647 (both from Jackson Immunoresearch, Suffolk, UK). Coverslips were mounted on glass slides with fluorescence mounting medium. Samples were analyzed using a Zeiss LSM510 laser confocal microscope (Carl Zeiss Ltd, Welwyn Garden City, UK) equipped with an argon (488-nm excitation line; 510 nm emission detection) laser and a diode-pulsed, solid-state laser (excitation, 561 nm; emission, 572-nm; long-pass filter).

### Western blotting

Western blotting was carried out as previously described [25]with some modifications. Briefly, cells were lysed in lysis buffer (50mM Tris.HCl, pH 6.8, 10% glycerol, 2% sodium dodecyl sulfate, 0.001% bromophenol blue, 100mM DTT, 10X protease inhibitor cocktail (Roche) and phosphatase inhibitors (Sigma)). Samples were heated to 95°C for 10 minutes and stored at -80°C. Lysates were subjected to SDS/PAGE on 15% Tris-Tricine Criterion (Bio-Rad, Hercules, CA) minigels followed by transfer to 0.2-μm-pore polyvinylidene difluoride membrane (Immun-Blot PDVF membrane; Bio-Rad). Membranes were probed with antibodies to mouse anti-human LC3 (2G6, nanoTools Antikörpertechnik GmbH, Teningen Germany) and mouse anti-human β–actin (Sigma). Following exposure to HRP-conjugated goat anti-mouse secondary antibody (Cell Signaling Technology, Danvers, MA, USA), proteins were visualized by enhanced chemiluminescence using Supersignal West Pico chemiluminescent substrate (ThermoScientific, Rockford, IL, USA).

### Measurement of NFκB Activity

THP-1 XBlue™ cells (Invivogen), which are stably transfected for a reporter gene for NFκB activation, were used to assess the activation of NFκB by PLGA microparticles. THP-1 Blue cells were cultured in RPMI-1640 with heat inactivated 10% FBS and 200μg/ml of Zeocin™. The cells were seeded at a final density of 1 x 10^6^ cells / ml in 96-well plates in the presence of 100nM PMA for 72 hours. The medium was changed each day for 5 days after differentiation to remove any residual PMA, which could lead to false positive results. After 5 days the cells were infected or treated with PLGA MPs of 0.8μm or 2.2μm and 1.8 μm PS MPs for 24 hours, after which time 20μl of medium was added to 180μl QUANTI-Blue™ and incubated for 4 hours. The SEAP (secreted embryonic alkaline phosphatase) activity was assayed using a Wallac (Perkin Elmer) plate reader at 650nm. LPS (100ng/ml) was used as a positive control for NFκB activation.

### Caspase activity assays

Fluorogenic caspase substrate assays for caspase-3/7, -8 and -9 were carried out using the substrates acetyl Asp-Glu-Val-Asp 7-amido-4-methylcoumarin (Ac-DEVD-AMC), Ac-Ile-Glu-Thr-Asp-AMC (Ac-IETD-AMC) and Ac-Leu-Glu-His-Asp-AMC (Ac-LEHD-AMC) (all from ENZO Life Sciences) respectively. THP-1 cells were seeded at a density of 1x10^5^ cells /ml as described in the cell culture section and both uninfected cells and cells infected with Mtb H37Ra (as described in the cell culture section) were treated with 2.2μm MPs for 1 hour and then cultured for a total of 24 hours. Extracts were prepared by lysing cell pellets collected at the indicated times in 75μl of lysis buffer (25mM HEPES (pH 7.5), 0.1% CHAPS, 1mM dithithreothiol) followed by 3 freeze thaw cycles at -80°C and centrifugation at 15,000g for 15 min. Aliquots of the cleared lysates were incubated in a microtitre plate with the fluorogenic substrate (final concentration of 80 μM) and incubated for 30 minutes at 37°C. Fluorescence intensity was detected on a Victor plate reader (Perkin Elmer) at 360nm excitation and 460nm emission. Samples were normalised for protein content using a BCA assay. Cells treated with staurosporine (200ng/ml) were used as a positive control for caspase activity.

### Treatment of macrophages with pharmacological inhibitors

Differentiated THP-1 cells were infected with H37Ra and then pre-treated with the pan caspase inhibitor ZVAD-fmk (Bachem) (50μM), the NFκB inhibitor SN50 (18μM) and the PI3 kinase inhibitor 3-MA (10mM) for 1 hour prior to treatment with 2.2μm PLGA MP. Fresh inhibitor was added after removal of unphagocytosed particles after 48 hours. Cells were incubated at 37°C for a total of 72 hours after which they were lysed and plated for enumeration on Middlebrook agar plates.

### Cytokine Secretion Assays

Supernatants from differentiated THP-1 cells with and without H37Ra infection were collected after 24 hours and analysed according to the manufacturer’s instructions using multiplex plates (Meso Scale Discovery) for the following 10 cytokines: IFN-γ, IL-10, IL-12 p70, IL-13, IL-1β, IL-2, IL-4, IL-5, IL-8, TNF-α. An additional single-plex ELISA was carried out for IL-6 using the same supernatants. The plates were read on a SECTOR Imager 2400 and analysed using DISCOVERY WORKBENCH assay analysis software (MSD).

### Statistical Analysis

Statistical analysis was carried out using the Students t-test or One Way Analysis of Variance followed by Tukeys Multiple Comparison test. A p-value of < 0.05 was taken to be statistically significant.

## Results

### Microparticle characterisation

The MP size used in this study was chosen based on previous results that established the optimal size to target alveolar macrophages both anatomically (via inhalation) and at a cellular level (via phagocytosis) is approximately 2μm [7]. Each batch of PLGA MPs manufactured was characterised for size, surface charge and morphology prior to further testing. The spherical MPs produced for this study had a mean geometric diameter of 2.2 ± 0.3μm and a ζ-potential of -33.4 ± 2.62 mV. When required batches of smaller 0.8 ± 0.1μm MPs were also manufactured by increasing the duration of homogenisation and characterised appropriately [7].

### Mycobacterial growth in macrophages is restricted by PLGA microparticles

**Fig 1:**
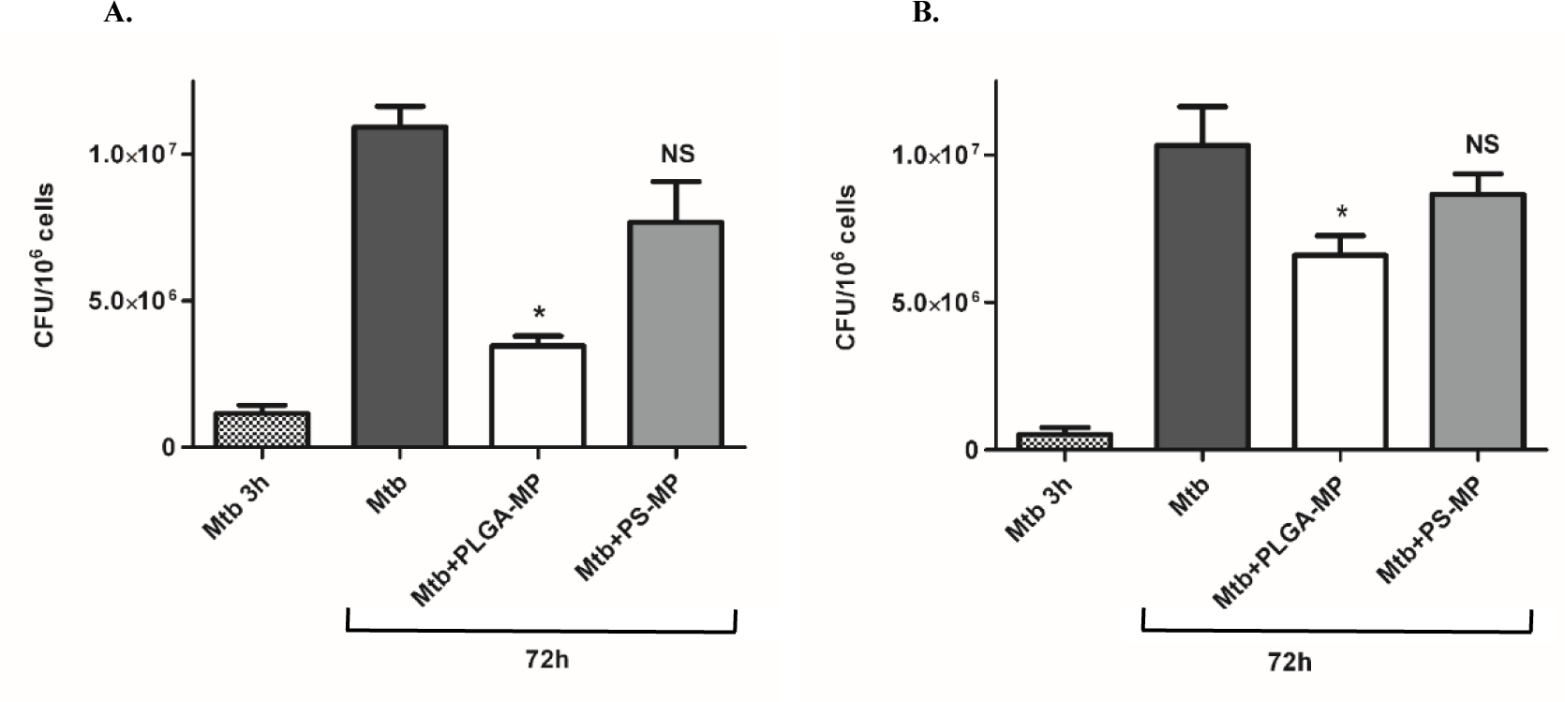
Drug-free PLGA MPs restrict the growth of Mtb in macrophages. PMA differentiated THP-1 cells were infected with (A) H37Ra or (B) H37Rv to reach an MOI of 1–5 phagocytosed bacilli per cell. The cells from several wells were lysed and serial dilutions were plated on Middlebrook 7H10 agar plates to determine the bacterial load at 3h (Mtb 3h). Cells in the remaining wells were left untreated (Mtb) or treated with 2.2μm PLGA MPs (Mtb+PLGA) or 1.8 μm polystyrene (PS) MPs (Mtb+PS) for 1 hour at 37°C, 5% CO_2_ after which the cells were washed and incubated for a further 72 hours. The cells were then lysed, combined with the medium and serial dilutions plated on Middlebrook 7H10 agar plates. The plates were incubated at 37°C for 14–21 days and colony forming units were counted. Results are expressed as mean +/- SEM, * p< 0.05 compared to untreated Mtb-infected cells. NS = not significant compared to untreated Mtb-infected cells.

Previous studies have shown that poly(lactic acid) (PLA) MPs can activate macrophages and enhance their ability to kill intracellular Mtb [26]. To determine whether MPs have an impact on Mtb growth in macrophages, THP-1 cells, infected with H37Ra or H37Rv for 3h, were treated with 200μg/ml of either 2.2 μm PLGA MPs or polystyrene (PS) MPs (1.8μm) for 1h, washed and cultured for a total of 4 days. The MP size and dose were chosen based on previous results that indicated that approximately 2 μm is the optimal size to target alveolar macrophages [7] and to reduce Mtb CFU by at least 5 log fold when loaded with rifampicin [8]. Treatment with drug-free PLGA MPs significantly reduced the CFU count of both strains of Mtb compared to that of untreated cells (p<0.05). PS MPs of a similar size did not have a statistically significant effect on growth of Mtb (Fig 1).

### The impact of microparticles on cell death and caspase activity

**Fig 2:**
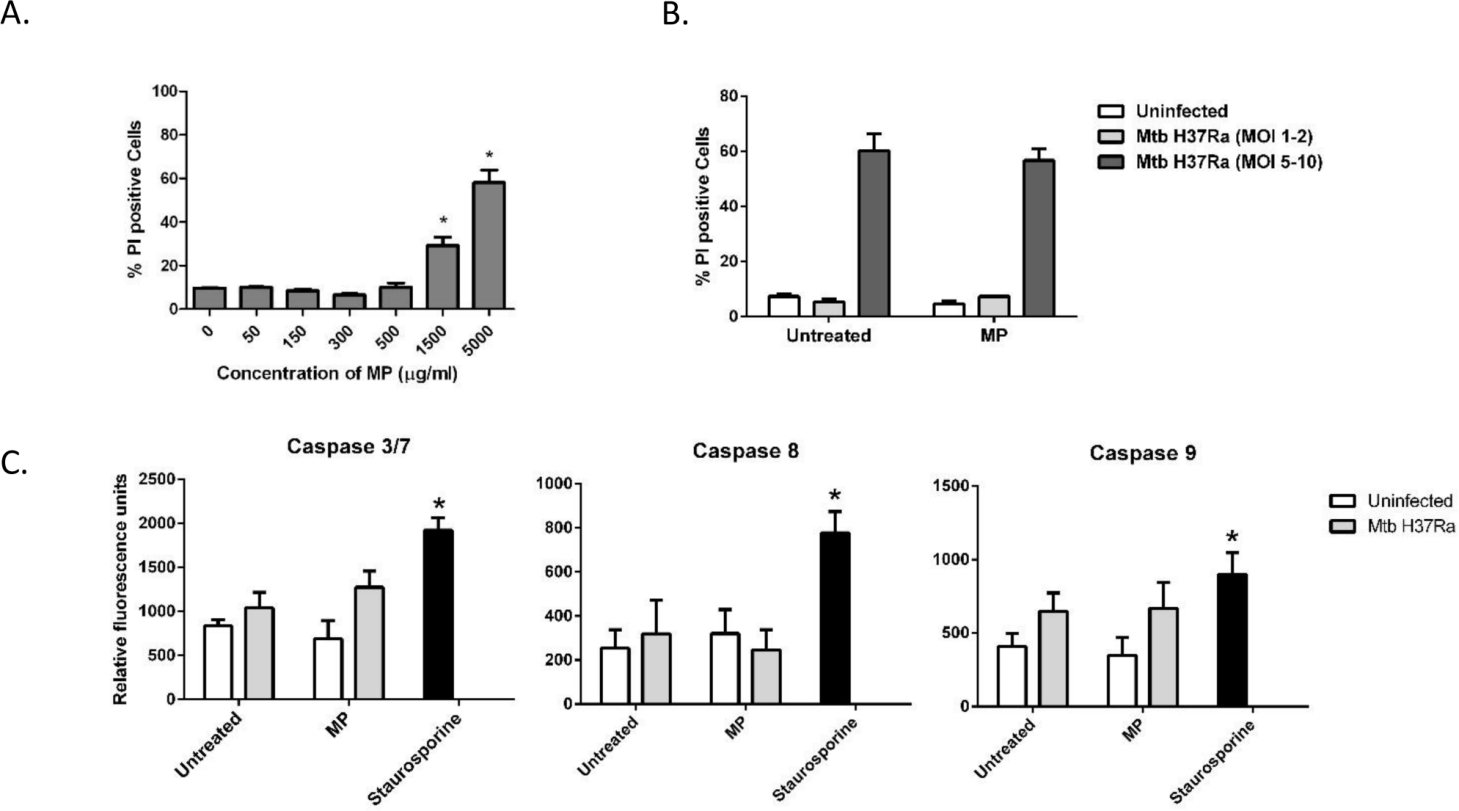
The effect of MPs on cell death and caspase activity in the presence or absence of Mtb infection. (A) PMA-differentiated THP-1 cells were treated with PLGA MPs at the indicated doses for 1 hour. The cells were washed to remove extracellular MPs and incubated for a total of 24 hours at 37°C, 5% CO_2._ Cell death was measured by propidium iodide exclusion assay and automated fluorescence microscopy. (B) THP-1 cells were uninfected or infected with Mtb (H37Ra) to reach an MOI of 1–5 phagocytosed bacilli per cell, washed and treated with 2.2μm PLGA MPs for 1 hour at 37°C, 5% CO_2_ after which the cells were washed and incubated for a total of 24 hours. Cell death was measured as above. (C) In parallel experiments cell lysates were assayed for caspase activity using caspase substrates coupled to AMC and proteolytic cleavage of the substrates was measured on a microplate reader (Ex/Em = 360/460nm). Staurosporine (0.4μg/ml) was used as a positive control for apoptosis. Results are expressed as arbitrary fluorescence units (mean +/- SEM) and are representative of 3 independent experiments, * p< 0.05 compared to uninfected cells.

Induction of macrophage apoptosis has been demonstrated to inhibit the growth of intracellular pathogens including Mtb [27, 28] whereas necrosis is not beneficial to the host in this regard [29]. The effect of MP treatment on macrophage viability was assessed by automated fluorescence microscopy using the propidium iodide exclusion assay. An initial experiment was carried out to determine whether PLGA-MPs were cytotoxic; macrophages were treated with increasing doses of MPs using the PI exclusion assay to determine the viability of the cells [25, 30]. There was no significant effect of MPs on viability until the concentration of MPs reached 1.5mg/ml (Fig 2A), which was 7.5 fold above the concentration used to inhibit Mtb growth (see Fig 1). All further experiments were carried out using the therapeutic dose of 200μg/ml. At this concentration of MPs the level of cell death in uninfected and Mtb infected cells was not significantly increased by treatment with PLGA MPs for 24 hours (Fig 2B).

Caspase activity is a hallmark of apoptosis. Others have shown that PLA MPs can activate caspases in Mtb-infected macrophages [21]. In the present study the ability of PLGA MPs to activate caspases in uninfected cells was evaluated by incubating cell lysates with fluorescently-labelled peptide substrates for caspase-3/7, -8 and -9 respectively. No significant increases in caspase activity were observed following treatment of macrophages with PLGA MPs alone for 24 hours (Fig 2C). To investigate whether PLGA MPs could increase the levels of apoptosis in Mtb-infected macrophages, infected cells were treated with MPs and assayed for caspase activity as above. Caspase activity in Mtb-infected cells was not significantly increased by the MPs (Fig 2C).

### Activation of NFκB by PLGA microparticles

**Fig 3:**
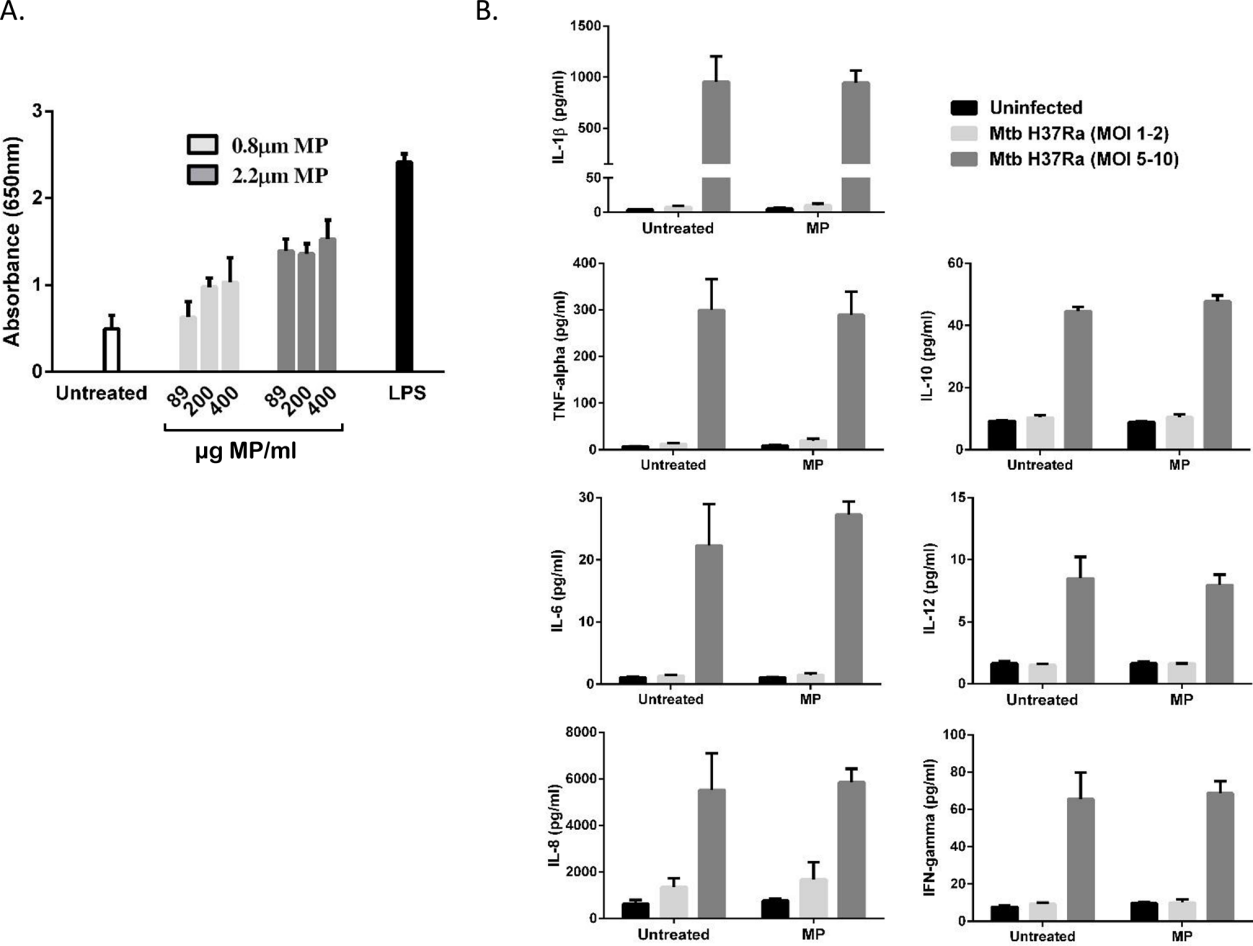
PLGA MPs activate NFkB but do not stimulate pro-inflammatory cytokine secretion by macrophages. (A) THP-1 XBlue™ cells were differentiated with PMA for 72h and the medium was changed for several days before treatment. Cells were then treated with 0.8μm or 2.2μm PLGA MPs at the indicated concentrations for 1 hour. Extracellular MPs were washed off and the cells were incubated at 37°C for a total of 24 hours. Supernatants were analysed for the presence of SEAP by means of a substrate reaction and the absorbance was measured at 650nm on a microplate plate reader to determine NFkB activation. Results shown are means +/1 SEM for three independent experiments. LPS (100ng/ml) treatment was used as a positive control for NFκB activation. (B) THP-1 cells were incubated with media alone (media) or infected with Mtb H37Ra for 3 hours. The cells were then washed to remove extracellular mycobacteria and were treated for 1 hour with 200μg/ml MPs. After washing to remove extracellular MPs the cells were cultured for a total of 24 hours after which supernatants were assayed for cytokine levels using MSD multiplex assays. Cytokine levels are reported as mean +/- SEM and are representative of three independent experiments performed in triplicate, * p< 0.05 compared to untreated control.

The potential for PLGA MPs to trigger endogenous macrophage responses was tested using a THP-1 Blue™ cell line to evaluate their ability to activate NFκB. The results shown in Fig 3A demonstrate that MP activation of NFκB occurred in a size dependent manner. A significant increase in NFκB activation is observed with the 2.2μm MPs (p<0.05). PS MP also activated NFkB (S1 Fig).

### Cytokine secretion profile of microparticle-treated macrophages

NFκB plays a pivotal role in innate immune signalling; its activation via TLR receptors can lead to the upregulation of a network of pro- and anti-inflammatory cytokine genes. These cytokines can influence intracellular growth of Mtb; for example, both TNF and IL-1β play a protective role in Mtb infection contributing to the bactericidal activity of macrophages whereas IL-10 promotes Mtb survival by preventing phagosome-lysosome fusion [31]. To determine whether PLGA MPs could induce cytokine secretion of uninfected THP-1 cells or alter the cytokine profile of Mtb-infected cells, THP-1 cells were untreated or treated with MPs as before. After 24h the medium was removed and a secretion profile of 11 cytokines was determined by MSD ELISA. Treatment of THP-1 macrophages with 2.2μm MPs did not result in any increase in cytokine secretion above background levels (Fig 3B). As expected, macrophages infected with Mtb secreted a range of pro and anti-inflammatory cytokines including IL-1β, TNF, IL-6 and IL-10 (Fig 3B). However, PLGA MP treatment did not alter the cytokine profile of Mtb-infected macrophages (Fig 3B). Levels of IL-2, IL-4, IL-5 and IL-13 were unaltered by infection in the presence or absence of Mtb.

### Induction of autophagy by PLGA microparticles

**Fig 4:**
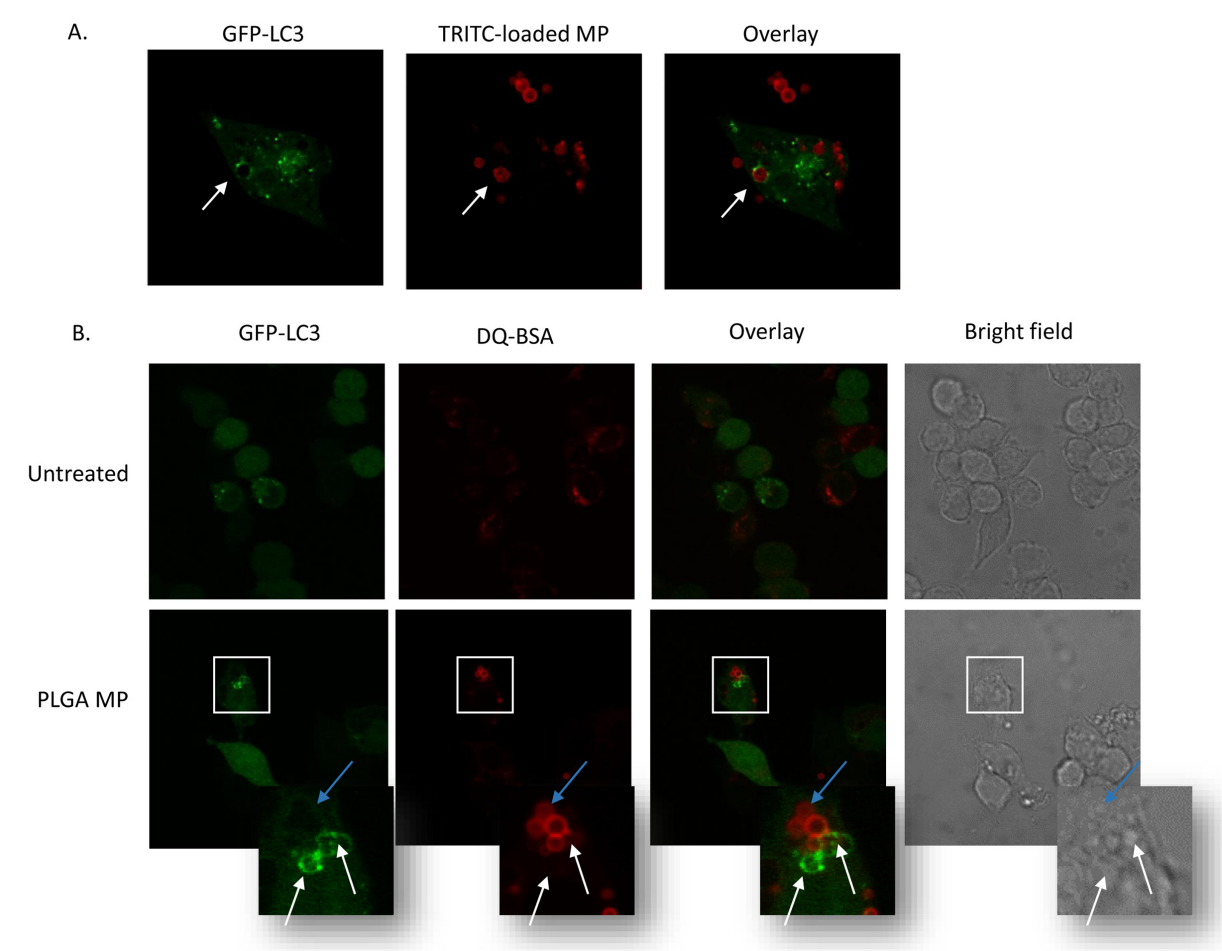
PLGA MPs co-localise with LC3 following phagocytosis by macrophages. Live confocal microscopy images of immortalized murine BMDMs expressing GFP-LC3 were acquired using a ×63 oil objective following incubation with (A) TRITC-loaded MPs (red) for 3h, or (B) pre-loaded with DQ-Red-BSA for 2 hours to visualize lysosomes, washed, rested for 1 hour in medium without DQ-Red-BSA and then incubated with blank PLGA MPs (200μg/ml) for 1h. The white arrows indicate localization of GFP-LC3 with MPs, blue arrows indicate localization of lysosomes with MPs. The results shown are representative of five independent experiments.

Autophagy is a mechanism whereby cells recycle cellular components by enclosing them in autophagosomes that fuse with lysosomes to mediate degradation of their contents. Autophagy also plays a role in host defence by engulfing and degrading microbes [32]. Mtb has evolved to arrest phagosome maturation by targeting PI3-kinase signalling and Rab-GTPases [33]. However, induction of autophagy, by starvation or rapamycin treatment, can overcome the phagolysosomal maturation block to degrade intracellular mycobacteria [34]. To test the ability of MPs to induce autophagy, immortalised murine BMDM stably expressing GFP- LC3 were treated with PLGA MPs and analysed by confocal microscopy. A lipidated form of LC3, LC3-II, is a marker of autophagosomes. In the absence of MPs the cells displayed diffuse cytoplasmic GFP staining with occasional puncta present. Live cell imaging showed accumulation of GFP- LC3 around MPs as they were being phagocytosed (Fig 4A and S1 Movie). PS MP also co-localized with GFP-LC3 (S1 Fig) in agreement with previously published results [35].

Although the formation of LC3-positive vesicles could be seen to occur during phagocytosis of MPs, quantitative analysis of puncta at various times after the addition of MPs did not reveal a significant difference in the number of puncta per cell compared to untreated cells. This suggests that the association of LC3 and MPs is transient and that LC3 positive phagosomes may fuse with lysosomes causing degradation of GFP- LC3 by hydrolytic enzymes. DQ-Red-BSA is a substrate of acidic hydrolases and a marker for lysosomes in live cells. After approximately 2 hours MPs had trafficked to DQ-Red-BSA positive lysosomes, the majority of which were not positive for GFP-LC3 (Fig 4B).

**Fig 5:**
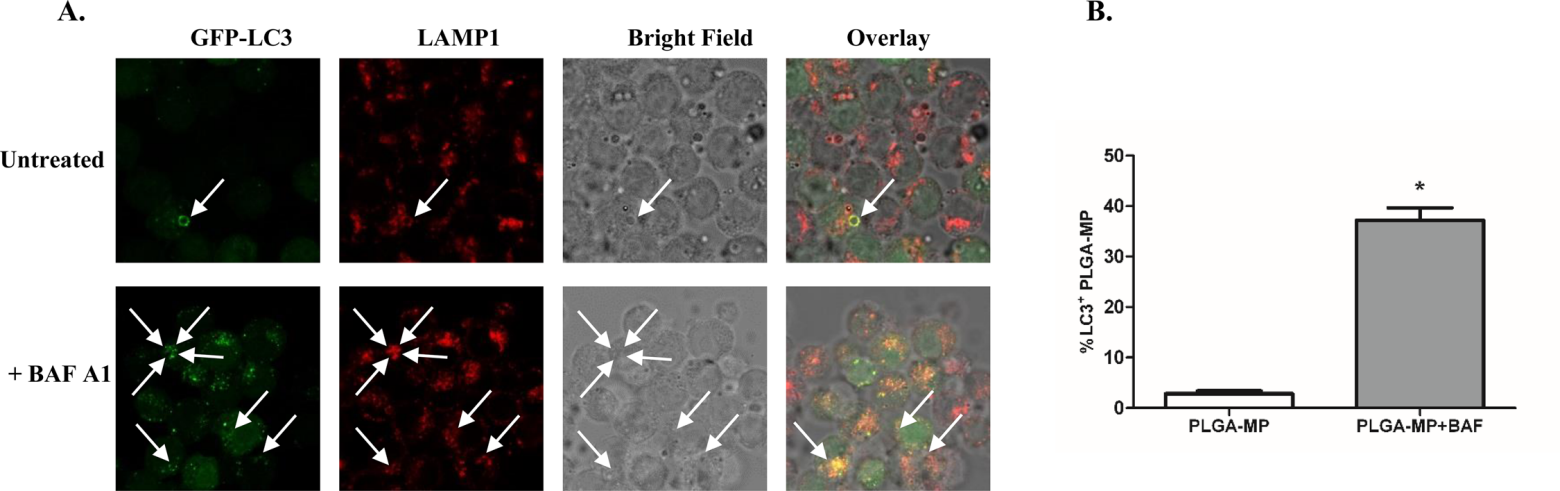
PLGA MPs phagocytosis triggers autophagic flux in macrophages. (A) Immortalized murine BMDMs expressing GFP-LC3 incubated with PLGA-MP for 3 hours in the presence of bafilomycin A1 (30nM) or vehicle (DMSO) were subjected to immunofluorescent staining for LAMP-1 and imaged by confocal microscopy to determine (B) the proportion of PLGA MP co-localising with GFP-LC3 which are reported as mean +/- SEM.of three independent experiments, * p< 0.05. The white arrows indicate localization of GFP-LC3 with PLGA MPs. LAMP1 staining was included to aid in the visualization of MP phagosomes.

To determine whether autophagic flux was taking place macrophages were incubated with PLGA MPs for 3 hours in the presence of the vacuolar H+ ATPase inhibitor bafilomycin A1 which prevents acidification of lysosomes or vehicle (DMSO). There was a significant difference in the proportion of PLGA MP associated with GFP-LC3 in bafilomycin A1-treated cells compared to cells which were untreated (Fig 5C).

### PLGA-MPs triggers autophagic flux in Mtb-infected macrophages

**Fig 6:**
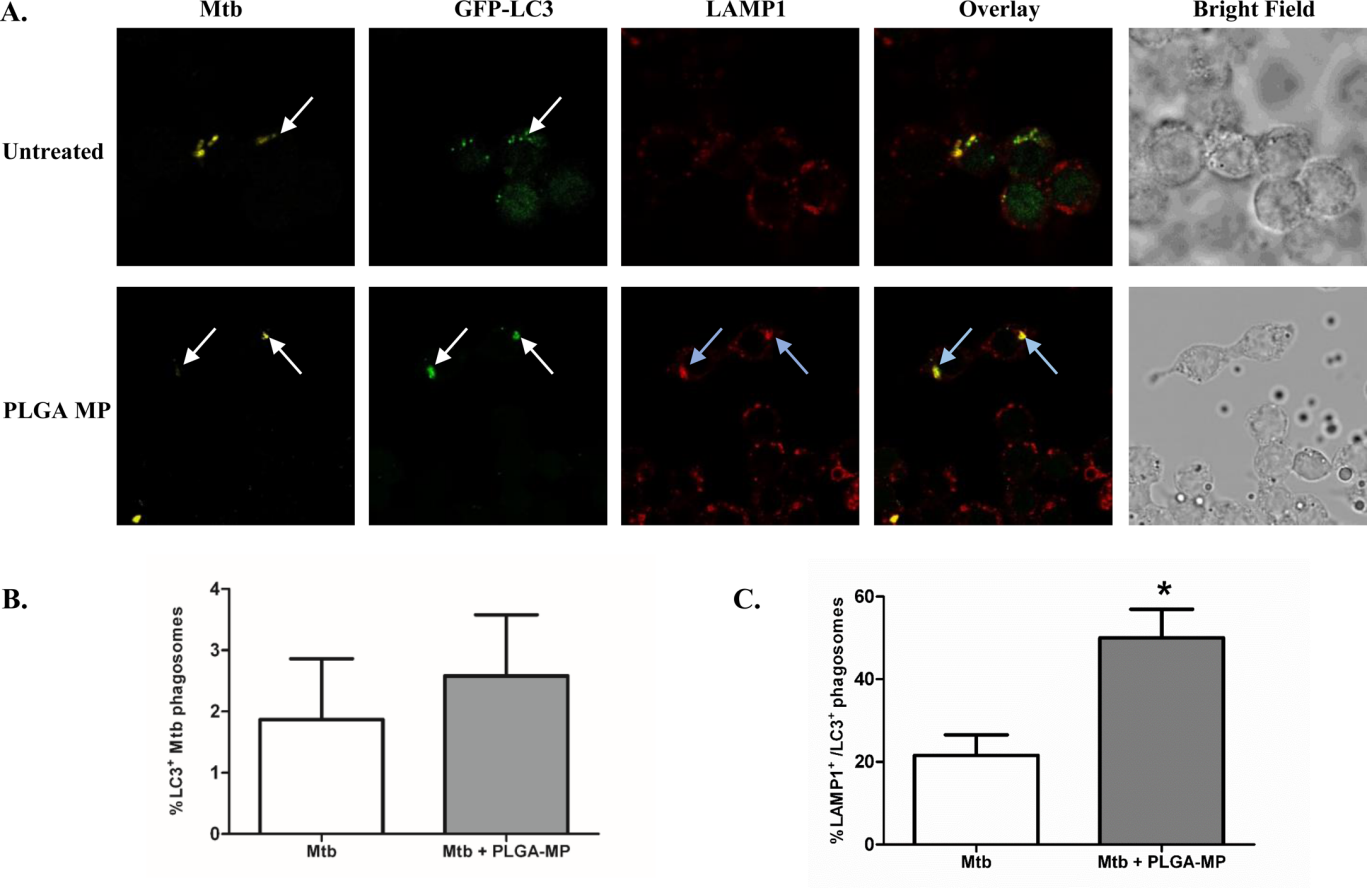
PLGA MPs trigger autophagic flux in Mtb-infected macrophages. Immortalized murine BMDMs expressing GFP-LC3 were infected with Mtb H37Ra at an MOI of 1–5 phagocytosed bacilli per cell followed by treatment with PLGA MP as before. (A) After a total of 24 hours, infected macrophages were fixed and stained with anti-LAMP1_and anti-Mtb antibodies, and then observed by laser scanning confocal microscopy. The proportion of (B) LC3 positive Mtb phagosomes and (C) LC3 positive phagosomes which were also positive for LAMP1 were counted. Data represent the mean +/- SEM of three independent experiments in which more than 100 phagosomes were counted for each condition. *P < 0.05. The white arrows indicate localization of Mtb phagosomes with GFP-LC3, blue arrows indicate co-localization of Mtb, GFP-LC3 and LAMP1. The results shown are the means of three independent experiments.

To assess the role of autophagy in PLGA-induced anti-mycobacterial activity of macrophages, Mtb-infected cells were incubated with PLGA-MP and examined by confocal microscopy after 24 hours (Fig 6A. The number of GFP-LC3 positive Mtb phagosomes was similar in untreated cells and those treated with PLGA-MP (Fig 6B). However, there was a significant increase in the number of GFP-LC3 positive phagosomes that were positive for the late endosomal/lysosomal marker LAMP-1 in macrophages treated with PLGA MP (Fig 6C).

### Blocking autophagy and NFκB reverses the inhibition of Mtb growth by microparticles

**Fig 7:**
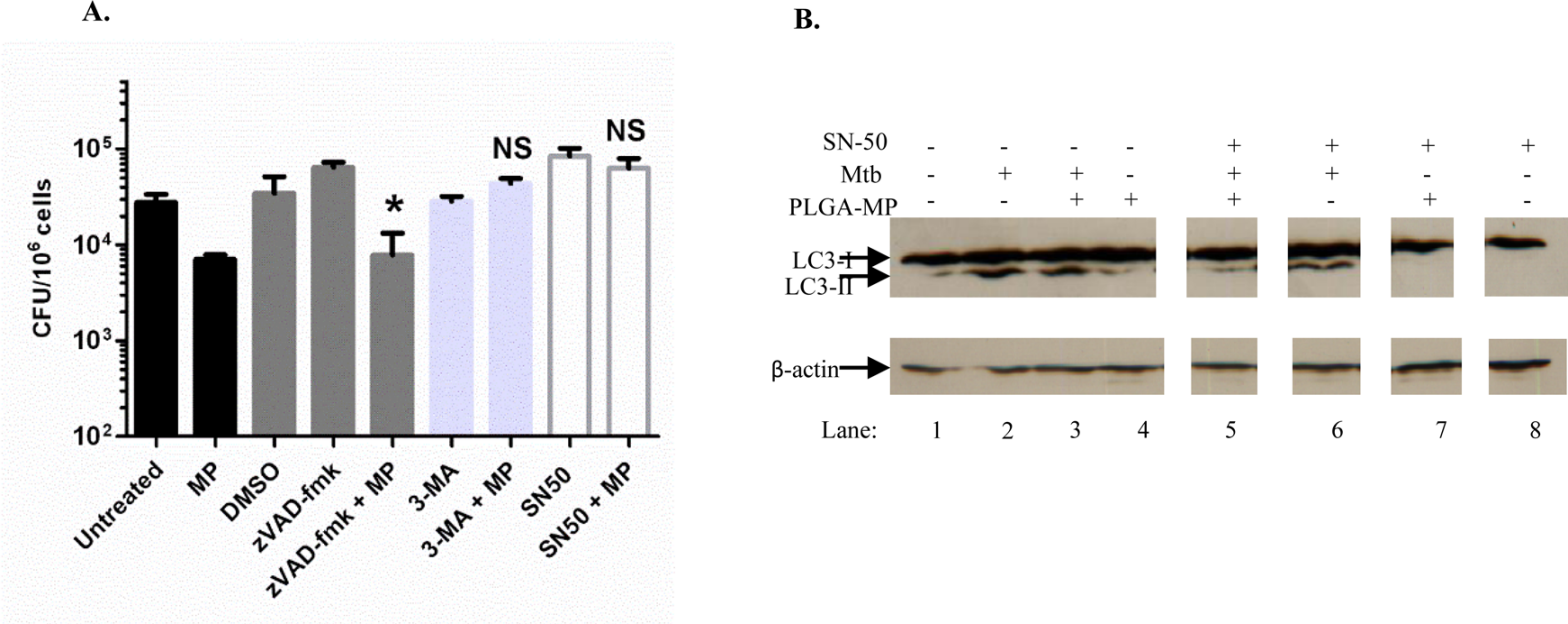
Blocking autophagy or NFkB activity interferes with killing of Mtb in PLGA PLGA MP-treated macrophages. (A) PMA-differentiated THP-1 cells were untreated or pre-incubated with inhibitors (50μM zVAD.fmk, 10mM 3-MA or 10μM SN50) vehicle (DMSO) for 1 hour and then infected for 3 hours with H37Ra at an MOI of 1–5 phagocytosed bacilli per cell. Extracellular mycobacteria were washed and the cells were incubated with 2.2 μm PLGA MPs (200μg/ml) for 1 hour followed by washing to remove extracellular MPs. The cells were then incubated for a total of 72 hours after which CFU were determined by serial dilution on Middlebrook agar plates. * p< 0.05 compared to untreated (for MPs) or DMSO (for zVAD-fmk). NS = not significant compared to untreated cells. (B) Induction of LC3-II in macrophages infected with H37Ra Mtb. Macrophages were infected with Mtb for 3 hours and treated with PLGA MP in the presence or absence of SN50 for a further 3 hours. Whole-cell lysates were subjected to SDS-PAGE, followed by western blot analysis using the indicated antibodies.

We next investigated the mechanism by which infected macrophages treated with PLGA MPs achieved a reduction in Mtb growth using pharmacological inhibitors. As previous results had shown that these MP delivery systems could induce NFκB activation (Fig 3A), a peptide inhibitor of NFκB, SN50, was used to test whether MPs could reduce Mtb viability when NFκB was inhibited. To confirm that SN50 inhibits NFκB activity THP-1 XBlue™ cells were incubated with the inhibitor and stimulated with LPS. As expected, LPS-induced NFκB activity was reduced in SN50-treated cells. Caspase activity was inhibited using the pan-caspase inhibitor zVAD.fmk to assess if cell death or inflammasome activation contributed to this mechanism. The effectiveness of zVAD.fmk at inhibiting caspase activity was confirmed by its ability to inhibit staurosporine-induced caspase 3/7 activity in THP-1 macrophages (S2 Fig). Finally, the PI3-kinase inhibitor 3-MA was used to inhibit autophagy. Inhibition of autophagy by 3-MA was confirmed by its ability to prevent the formation of LC3-positive puncta caused by uptake of zymosan particles (S2 Fig). As reported above (Fig 1A) macrophages infected with H37Ra and treated with PLGA MPs harboured significantly less Mtb 72 hours after the initial infection compared to untreated infected cells. In the presence of the caspase inhibitor z-VAD-fmk the MPs were still able to reduce Mtb CFU in a significant manner (p<0.05). Conversely, both NFκB and autophagy inhibition resulted in the inability of the MPs to reduce intracellular Mtb growth, indicating that both of these pathways play a role in the control of Mtb proliferation by macrophages (Fig 7A). As expected, there was no detectable increase in LC3-II protein levels in PLGA MP-treated cells above those of untreated cells when macrophage lysates were subjected to western blotting Fig 7B, lane 4). However, SN50 inhibited lipidation of LC3-I to LC3-II in PLGA MP-treated macrophages infected with Mtb (Fig 7B) suggesting that NFκB stimulates autophagy in Mtb-infected cells in the presence of PLGA-MP.

## Discussion

In this study we have shown that treatment of infected macrophages with 2.2μm PLGA MPs significantly reduces the intracellular burden of Mtb, while polystyrene MPs of a similar size did not. We hypothesised that PLGA MPs might augment the bactericidal activity of macrophages by activating innate immune pathways in macrophages as was previously demonstrated for PLA MPs [16, 26]. To test this hypothesis we examined the effect of PLGA MPs on a number of innate immune defence pathways including cell death, NFκB activity, cytokine secretion and autophagy. The bactericidal activity of the MPs was not associated with the induction of cell death or significant alterations in caspase activity. Nor did treatment of macrophages with MPs stimulate the secretion of inflammatory cytokines in the absence of infection or significantly alter the cytokine profile of Mtb-infected macrophages. However, phagocytosis of MPs was associated with activation of innate immune pathways as evidenced by increased activation of NFκB and the induction of autophagy. Inhibition of autophagy and NFκB abrogated the bactericidal effect of PLGA MPs in macrophages.

There has been much debate in the literature as to whether PLGA MPs are biologically active in the absence of a drug load with some studies showing that they are inert [10–12] while others have shown potent biological effects [13–18, 26, 36]. Indeed differential effects might be expected depending on the size, morphology and route of exposure of the particles. We therefore focused our studies (specifically for their application in inhaled treatment of Mtb) on PLGA particles of a very specific size and morphology (required for inhalation and cellular targeting) in relevant airway cells. NFkB is a master regulator of innate immune signalling pathways driving increased expression of pro-inflammatory genes such as NLRP3, TNF and IL-1β. In the present study, treatment with PLGA MPs increased NFkB activity in uninfected THP-1 cells. This is in agreement with a previous study which demonstrated that 5–7μm PLGA MPs activate NFkB in murine J774 macrophages [36]. However, in contrast to that and several other previously published reports [14–18, 26, 36], we found that PLGA MPs did not induce the secretion of either pro- or anti-inflammatory cytokines by uninfected macrophages. In addition, treatment with MPs did not significantly alter the cytokine profile of Mtb-infected macrophages ruling out a synergistic enhancement of TLR-mediated cytokine production by polymeric MPs in these cells [19]. These variations in outcome may be due to differences in particle size, composition or the responding cell lines. Despite its failure to drive cytokine secretion in THP-1 macrophages, inhibition of NFkB reversed the anti-mycobacterial effect of MPs, indicating that it plays a role in this process. NFkB activity regulates other stress-response pathways in macrophages including the modulation of autophagic flux. Indeed, in the present study inhibition of NFkB led to a reduction in the levels of LC3-II in infected macrophages treated with PLGA MP suggesting that it drives autophagy in this setting. The role that NFkB plays in autophagy is cell and context-dependent and the interplay between these two pathways is quite complex [37]. In the setting of mycobacterial infection, NFkB can drive increased expression of DNA-regulated autophagy modulator 1 (DRAM1) which is required for the initiation of anti-mycobacterial autophagy and promotes the fusion of autophagosomes with lysosomes [38]. Further investigation will be required to determine the mechanism of NFkB activation by PLGA MPs and its role in activating anti-mycobacterial pathways.

The induction of canonical autophagy by starvation or rapamycin treatment of Mtb-infected macrophages enhances their ability to control intracellular Mtb growth by overcoming a block in phagolysosomal maturation [34]. There is substantial evidence that biopersistent nanoparticles can cause autophagy [39] but little is known about the interaction of biodegradable MPs with the autophagic pathway. Phagocytosis of polystyrene MPs involves a non-canonical form of autophagy termed LC3-dependant phagocytosis (LAP) which results in translocation of LC3 to the phagocytic cup and the formation of single-membrane autophagosomes, in the presence [40] or absence of TLR agonists [35]. LAP links phagocytosis of synthetic particles by scavenger receptors such as MARCO (which can mediate the uptake of PLGA MPs *in vivo)* [13] to innate immune activation pathways in APCs [41]. We observed transient GFP-LC3 co-localization with particles during phagocytosis of PLGA MPs after which MPs trafficked to lysosomes. In addition, treatment with PLGA MPs increased autophagic flux in macrophages infected with H37Ra. Furthermore, the PI3-kinase inhibitor 3-MA abrogated the anti-mycobacterial activity induced by PLGA MPs, confirming a role for autophagy in this process. A detailed analysis of the autophagy pathway induced by PLGA MPs will be required to establish whether there is a link between LAP and the restriction of Mtb growth.

Our study has several limitations which should be kept in mind when interpreting the results. Firstly, although we show that PLGA MPs inhibited the growth of H37Ra and H37Rv, our mechanistic results on the involvement of autophagy and NFkB activity were obtained with the attenuated H37Ra strain of Mtb and may not apply to the macrophage response to virulent strains, However, both H37Ra and H37Rv inhibit phagosome acidification which can be overcome by inducers of autophagy [34]. Therefore, it seems reasonable to assume that PLGA MPs could have similar effects in macrophages infected with virulent H37Rv. Secondly, although non-biodegradable PS MPs also activated NFkB and triggered LAP in macrophages, they did not significantly affect intracellular growth of Mtb. The reasons for this are not clear but may be related to the different physico-chemical properties of these two materials. While polystyrene is inert and non-biodegradable, PLGA is hydrolysed to lactic and glycolic acid monomers which can be further catabolised *via* the tricarboxylic acid cycle. It is possible that PLGA or its metabolites influence innate immune pathways, in ways which are not yet clear, to contribute to the microbicidal activity of infected macrophages.

In addition to their potential use in inhaled therapies for TB, there is increasing interest in the use of biodegradable engineered particles for other biomedical applications. Polymeric MPs have been tested as delivery vehicles for subunit vaccines in order to improve their immunogenicity. As well as directly killing intracellular pathogens, autophagy also plays an important role in antigen presentation via the MHC-I and MHC-II pathways. Synthetic MPs are particularly efficient at cross priming antigen specific CD8^+^ T cells, which are important in defence against intracellular pathogens [42] and a target of anti-cancer vaccines [43]. This may be due in part to delivery of the antigen and adjuvant to the same endosomal compartment [44]. However, several studies have demonstrated that antigens encapsulated in PLGA MPs elicit more potent immunogenic responses than the corresponding soluble formulations [44, 45] even in the absence of an adjuvant [46]. Autophagy induced by PLGA MPs may contribute to the adjuvant effect of MPs *in vivo* by promoting LAP to enable efficient phagocytosis and subsequent delivery to endosomes and/or improve processing of antigens for presentation on MHC molecules.

Macrophage apoptosis restricts the intracellular survival of mycobacteria [47]. Yadev *et al* observed increased caspase activity and cell death in Mtb infected macrophages treated with PLA MPs [21]. PLGA MPs have also been shown to cause caspase-3-dependent apoptosis of circulating inflammatory monocytes in mouse models of inflammation [13]. In the present study we found that PLGA MPs did not cause a significant increase in caspase activity or induce apoptosis of either uninfected or infected macrophages at a dose at which they effectively inhibit Mtb growth. The lack of effect of the pan-caspase inhibitor zVAD-fmk on Mtb growth confirmed that apoptosis did not play a significant role in the ability of PLGA MPs to modulate Mtb replication in macrophages. This does not rule out the possibility that higher concentrations of PLGA MPs, which we found caused a significant amount of cell death (Fig 2A), could mediate mycobacterial killing by triggering apoptosis or another form of cell death.

While further long term testing is needed to fully evaluate the effects of MPs on inflammatory and immune pathways post inhalation, the results herein provide important information on the response of airway cells to inhaled PLGA MPs. This information is critical for the scientific, clinical and regulatory communities if the full potential of these advanced drug delivery systems is to be realised. Taken together our data indicate that 2.2μm PLGA MPs do not promote an inflammatory response from macrophages or lead to excessive cell death. They do, however, support the anti-mycobacterial activity of Mtb infected macrophages by inducing autophagy. This autophagy-inducing property of MPs acting in synergy with their anti-tubercular chemotherapeutic cargo may contribute to the efficacy of inhaled MPs in the treatment of TB.

## Supporting Information

S1 Fig PS-MP induce NFkB activation and autophagy. (A) NFkB activation was determined in the presence of PS-MP in THP-1 X Blue™ cells by measuring secreted embryonic alkaline phosphatase activity and (B) GFP-LC3 positive puncta (green) induced by PS-MP in the presence of Bafilomycin A1 (30nM) were visualised in live BMDB using an epifluorescence microscope (Olympus).
(TIF)

S2 Fig Validation of the efficacy of inhibitors. (A) Caspase 3/7 activity was measured in THP-1 macrophages the presence of the apoptotic inducer staurosporine (stauro) with or without zVAD.fmk (50μm). (B) NFkB activation by LPS (100ng/ml) in THP-1 XBlue™ cells was determined in the presence or absence of SN50 (18μM) by measuring secreted embryonic alkaline phosphatase activity in triplicate (* p< 0.05 compared to LPS alone) and (C) GFP-LC3 positive puncta (green) induced in BMDM by zymosan in the presence or absence of 3-MA (10mM), were visualised using an epifluorescence microscope (Olympus). Nuclei were counter stained with Hoechst 33358.
(TIF)

S3 Fig Full scan of LC3 western blot shown in Fig 7B. (TIF)

S4 Fig Full scan of β-actin western blot shown in Fig 7B. (TIF)

S1 Movie Co-localization of PLGA microparticle phagosomes with GFP-tagged LC3. Time lapse movie of immortalized murine BMDM expressing GFP-tagged LC3 showing phagocytosis of TRITC-labelled PLGA MPs. Time-lapse confocal images were obtained using a laser-scanning confocal microscope (LSM710; Carl Zeiss). Frames were taken every 12.6 s for 4 min.
(AVI)
