# Supplementary figures and images for: Treatment of Mycobacterium tuberculosis-Infected Macrophages with Poly(Lactic-Co-Glycolic Acid) Microparticles Drives NFκB and Autophagy Dependent Bacillary Killing
Source: PLoS One. 2016 Feb 19;11(2):e0149167. doi: 10.1371/journal.pone.0149167 (PMC4760758; doi:10.1371/journal.pone.0149167)

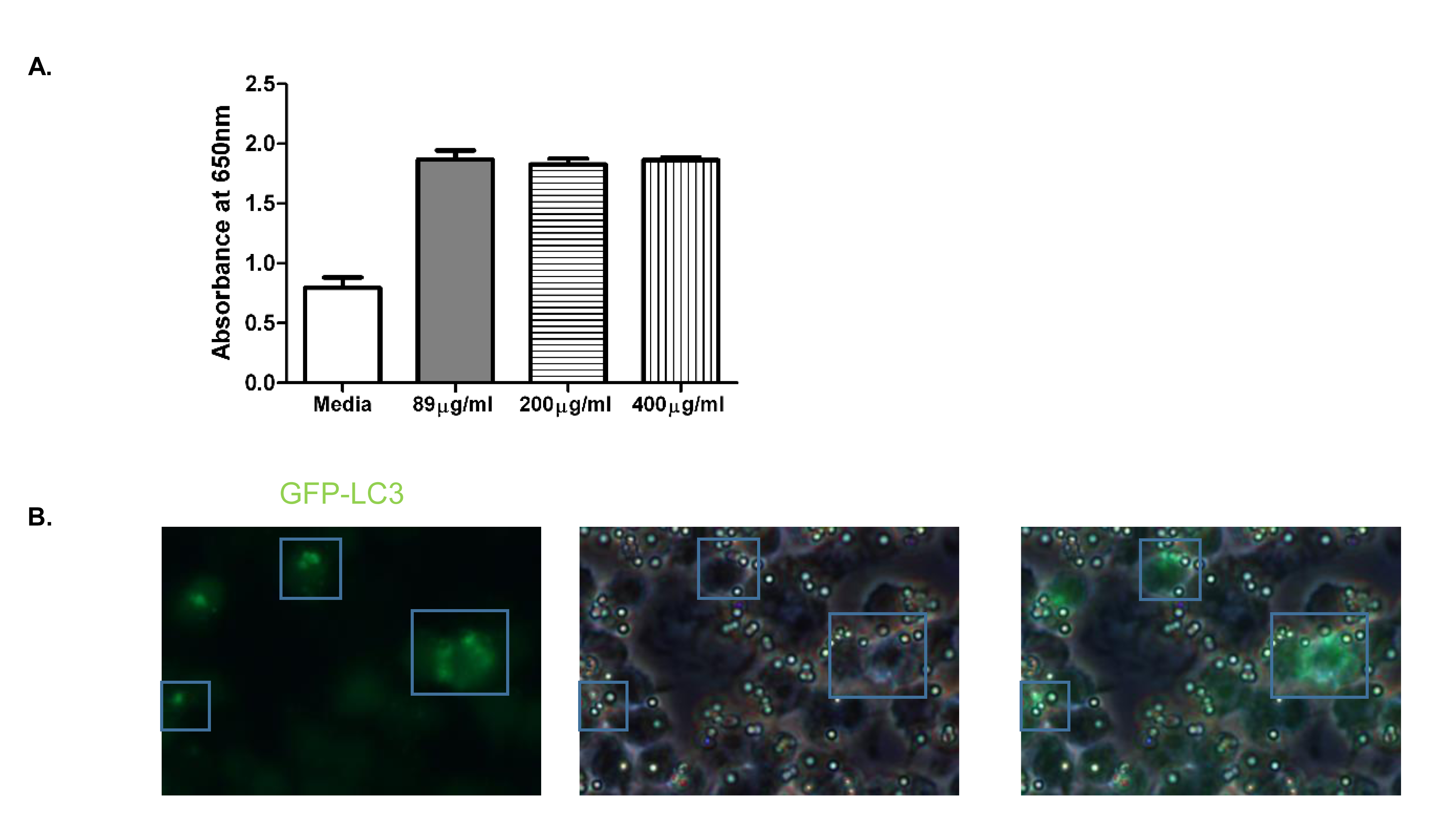

Supplement: S1 Fig — (A) NFkB activation was determined in the presence of PS-MP in THP-1 X Blue™ cells by measuring secreted embryonic alkaline phosphatase activity and (B) GFP-LC3 positive puncta (green) induced by PS-MP in the presence of Bafilomycin A1 (30nM) were visualised in live BMDB using an epifluorescence microscope (Olympus). (TIF) [file pone.0149167.s001.tif]

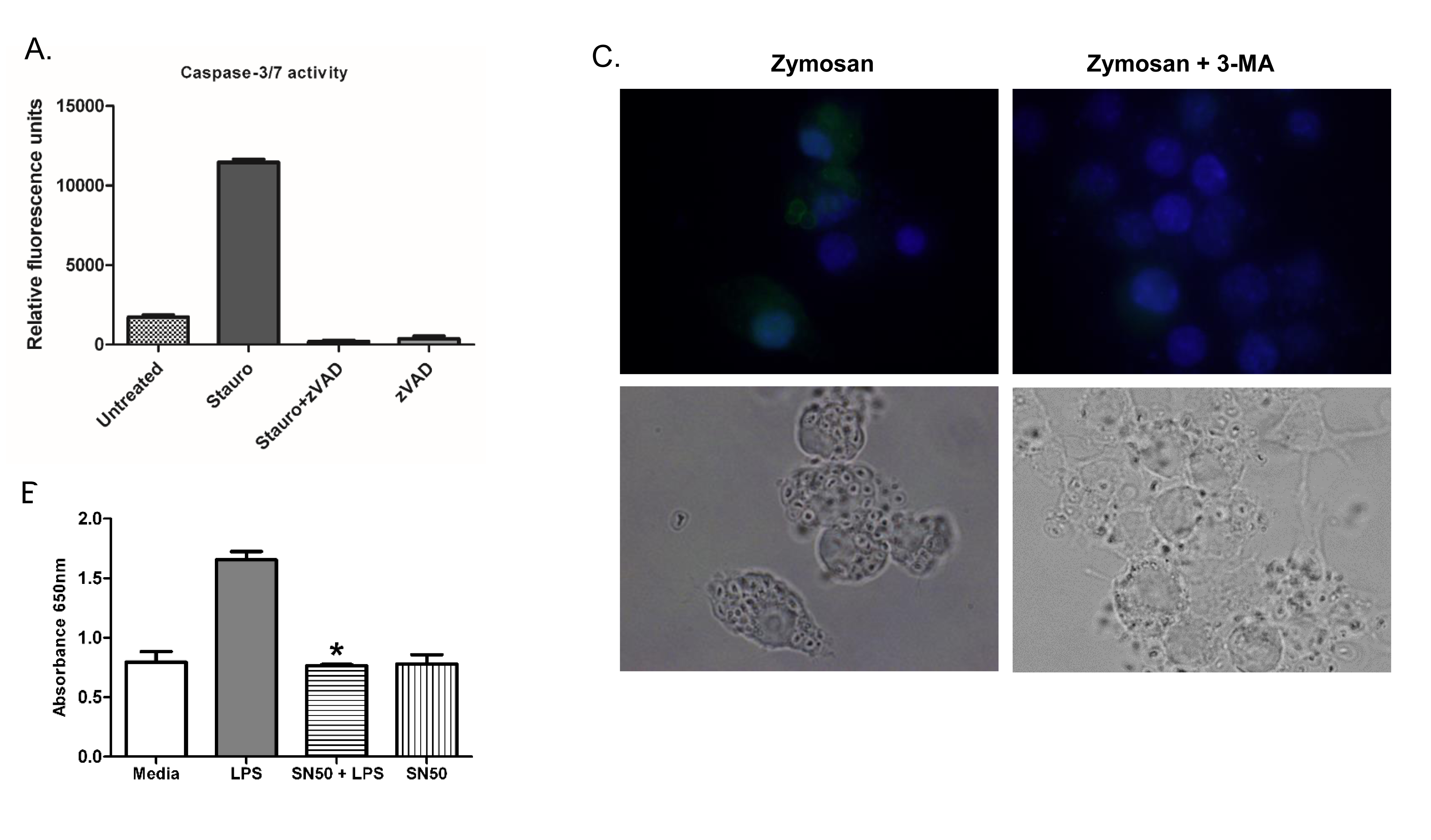

Supplement: S2 Fig — (A) Caspase 3/7 activity was measured in THP-1 macrophages the presence of the apoptotic inducer staurosporine (stauro) with or without zVAD.fmk (50μm). (B) NFkB activation by LPS (100ng/ml) in THP-1 XBlue™ cells was determined in the presence or absence of SN50 (18μM) by measuring secreted embryonic alkaline phosphatase activity in triplicate (* p< 0.05 compared to LPS alone) and (C) GFP-LC3 positive puncta (green) induced in BMDM by zymosan in the presence or absence of 3-MA (10mM), were visualised using an epifluorescence microscope (Olympus). Nuclei were counter stained with Hoechst 33358. (TIF) [file pone.0149167.s002.tif]

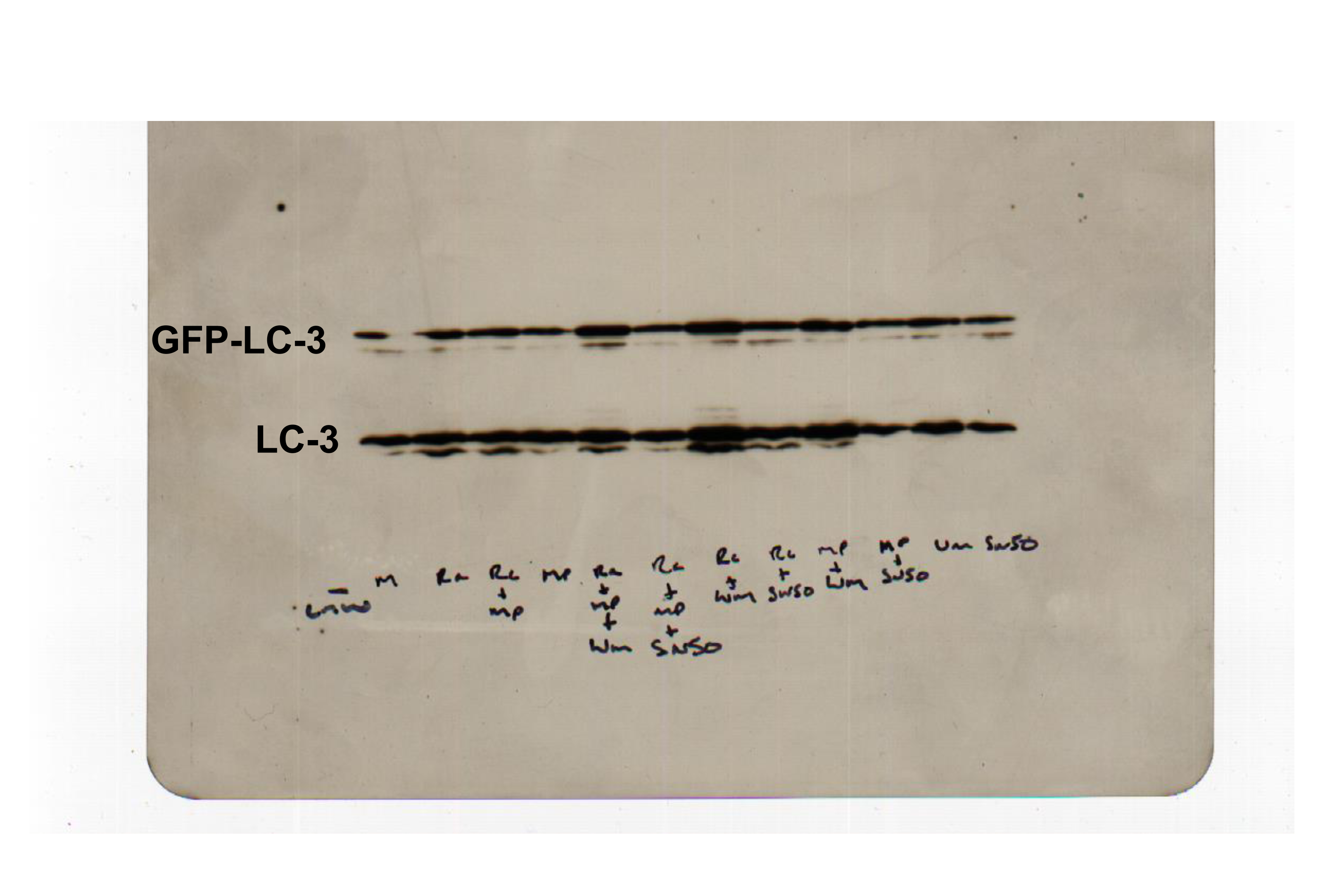

Supplement: S3 Fig — (TIF) [file pone.0149167.s003.tif]

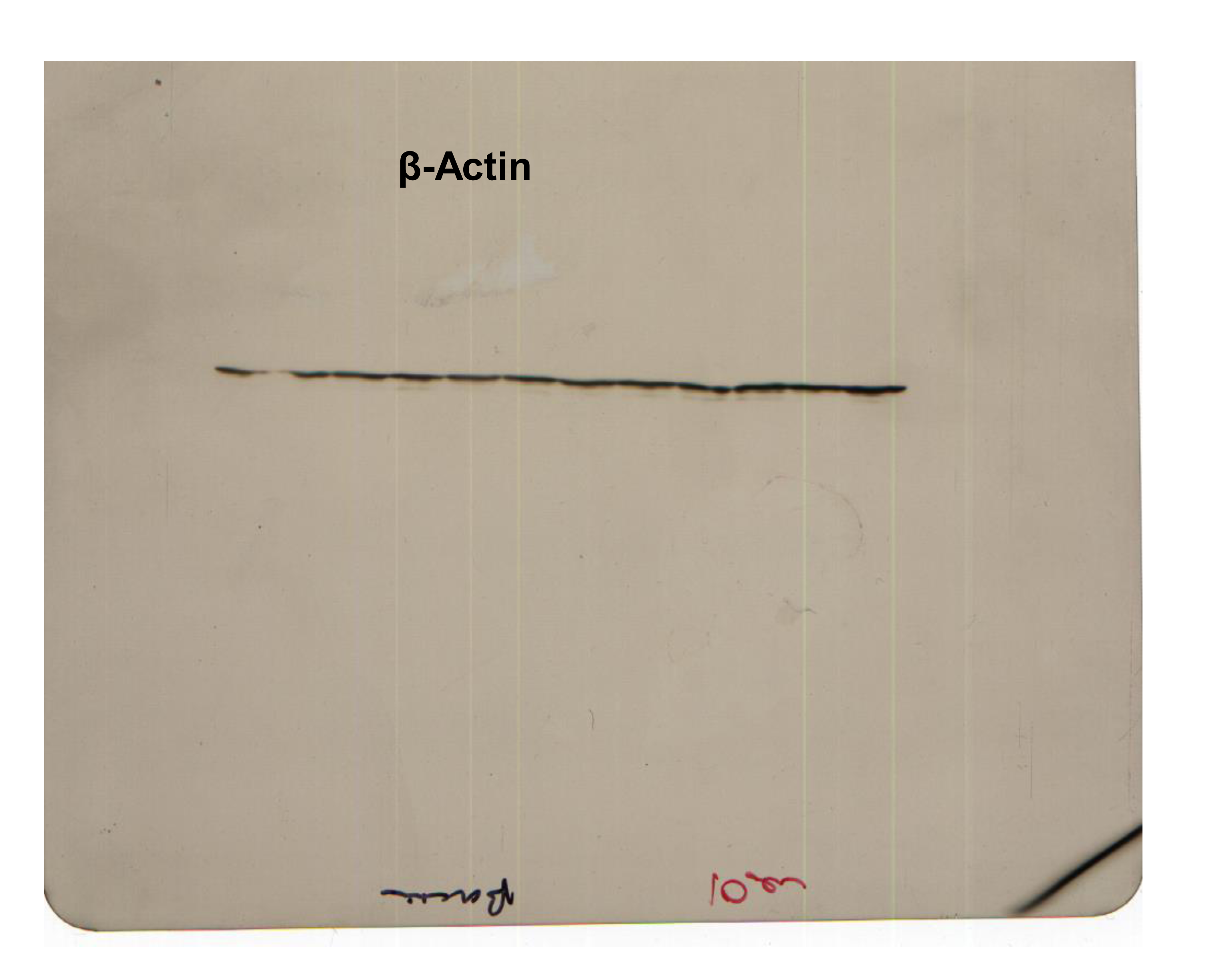

Supplement: S4 Fig — (TIF) [file pone.0149167.s004.tif]
